# Correction: Coumarin derivatives inhibit the aggregation of β-lactoglobulin

**DOI:** 10.1039/d2ra90064e

**Published:** 2022-06-29

**Authors:** Hasan Parvej, Shahnaz Begum, Ramkrishna Dalui, Swarnali Paul, Barun Mandal, Subrata Sardar, Nayim Sepay, Gourhari Maiti, Umesh Chandra Halder

**Affiliations:** Department of Chemistry, Jadavpur University Kolkata 700032 India uhalder2002@yahoo.com; Department of Chemistry, Lady Brabourne College Kolkata 700017 India

## Abstract

Correction for ‘Coumarin derivatives inhibit the aggregation of β-lactoglobulin’ by Hasan Parvej *et al.*, *RSC Adv.*, 2022, **12**, 17020–17028, https://doi.org/10.1039/D2RA01029A.

The authors regret that the name of the author Barun Mandal was incorrectly listed as Barun Mondal in the original manuscript.

The Royal Society of Chemistry apologises for these errors and any consequent inconvenience to authors and readers.

## Supplementary Material

